# Evaluating Radiation Exposures and Establishments of National Diagnostic Reference Levels for Patients Undergoing Chest Computed Tomography Examinations in Ethiopia

**DOI:** 10.1155/rrp/8206399

**Published:** 2025-03-27

**Authors:** Solomon Getachew Mekonnen, Seife Teferi Dellie, Daniel Zewdneh Solomon, Teklehaimanot Mezgebe Nguse

**Affiliations:** ^1^Department of Technology and Innovation Management, Adama Science and Technology University, Adama, Ethiopia; ^2^Department of Radiology, College of Health Sciences, Addis Ababa University, Addis Ababa, Ethiopia; ^3^Department of Radiography, School of Medicine, College of Health Sciences, Addis Ababa University, Addis Ababa, Ethiopia

**Keywords:** chest computed tomography, computed tomography dose index volume, dose length product, Ethiopia, national diagnostic reference levels

## Abstract

Computed tomography is invaluable for both diagnostic and therapeutic purposes. The common challenge is using an optimized CT technique to produce qualified images while giving patients the least amount of radiation possible. The objective of the study was to determine the national DRL values for adult patients undergoing chest computed tomography examination in Ethiopia. This was a retrospective cross-sectional study conducted in twenty three (23) different CT scan facilities on 801 patients who underwent chest computed tomography examinations in Ethiopia, in which participants were recruited by systematic random sampling. Data processing in this study was carried out with a quantitative analysis technique, namely descriptive statistics. The study variables were CTDI volume and dose length product (DLP) for the radiation doses for chest CT examinations. The age range for all adult patients was above 15 years old. Their body weights were in the range of 40–80 kg. The third quartile of the distributions of the median values of these variables was used to establish chest national diagnostic reference levels. The national DRL was compared with DRLs of other countries. Microsoft Excel form and SPSS software version 26 were used to collect and analyze survey data. A total of 801 patients were examined with an average age of 48.96 years. The patients were examined with their radiological department protocol using multislice CT (MSCT) from different manufacturers. For adult chest computed tomography examinations, the calculated dose length product and computed tomography dose index third quartile values that were used as national diagnostic reference levels were 512.9 mGy cm and 10.165 mGy, respectively.Even though the computed tomography dose index volume of the current study is less than that of all African and non-African countries selected for comparison, the proposed national DLP of the current study values were intermediate and substantially higher than those reported in similar studies from African and non-African countries, respectively. It is plausible to believe that the number of sequences, scan parameters, and automatic exposure control all contribute to better optimization and increased scanner dose efficiency for non-African countries, which is the cause of this discrepancy.

## 1. Introduction

Because of its exceptional anatomical details, high temporal and spatial resolution, and cross-sectional imaging capabilities, computed tomography (CT) has emerged as a crucial diagnostic imaging tool in numerous clinical settings. Since the early 1970s, CT scanners have been used in diagnostic radiology and have grown in popularity all over the world because of the significant and potentially life-saving clinical benefits it offers to patients [1, 2]. Compared to more popular conventional x-ray imaging procedures, CT requires higher radiation doses [3, 4]. The type of scanner, filtration, scan duration, thickness of the body, exposure parameters, and the imaging protocols are all factors that affect the CT radiation dose [5].

Since its introduction into diagnostic radiology in the early 1970s, CT scanners have become increasingly popular due to their significant and potentially life-saving clinical benefits for patients [6, 7]. Compared to more popular, traditional X-ray imaging procedures, CT requires higher radiation dosages by design [4, 8]. The CT radiation dose depends on a number of variables, such as the imaging protocols, exposure parameters, body thickness, filtration, scan time, and type of scanner [9, 10]. Diagnostic reference levels (DRLs) are operational tools for optimizing patients in diagnostic imaging as per the recommendation of the International Commission on Radiological Safety [11–13]. The DRLs are used to identify imaging procedures that result in abnormally high doses to patients; as a result, they should be reviewed for optimization, and if necessary, corrective action should be taken [10]. In order to optimize patient protection in CT, examination-specific scan protocols that are customized to the patient's age or size, region of imaging, and clinical indication must be applied to ensure that the dose to each patient is as low as reasonably achievable (ALARA) for the clinical purpose [14].

International organizations encourage the optimization of CT examination by comparing patient doses to establish DRL to be used as the indicator [15–17]. They also provide guidance for dose optimization and ensure justification of applicable dose for a given clinical indication. The volume CT dose index (CTDIvol), measured in milli-gray (mGy), and the dose-length product (DLP), measured in milli-gray-centimeter (mGy.cm), are the standard CT measurements used to set up DRLs [12, 18].

The radiation protection culture in most radiology facilities in Ethiopia is not well developed. This factor, together with the absence of proper maintenance and shelf life of the equipment, may increase the dose burden. The dosage of a patient can be managed by appropriate selection of parameters such as exposure factors, patient positioning, and examination protocol [12]. However, the level of proper understanding of the installed equipment is a big concern. In addition, in Ethiopia, radiographers' theoretical understanding of radiation protection does not consistently translate into actual practice. The possible risk of radiation exposure to the patient put emphasis on the need to establish DRLs for common radiologic examinations.

Many studies on CT DRLs have recently been carried out in various nations, and some of them have been updated successively [19–22]. The number of CT scanners used for medical purposes is growing at a faster rate in Ethiopia. The majority of CT exams follow the manufacturer's recommended protocols, which leads to multiphase protocols and insufficient professional effort to create local optimal conditions appropriate for specific patient needs and indications [23, 24].

Ethiopia lacks well-established national diagnostic reference levels (NDRLs), which means that patient radiation exposure is not appropriately managed. As a result, optimization, patient dose monitoring, record keeping, analysis, and high dose facility tracking are required. The dose quantities, such as CTDIvol and DLP, that CT scanners currently display to characterize radiation exposure to CT patients are not always recorded or used to track CT practice optimization. Consequently, the goals of this study—the first of its kind in Ethiopia—are to establish NDRLs for chest CT exams.

## 2. Materials and Methods

### 2.1. Study Area

With 12 national regional states, 2 chartered administrative cities under the Federal Democratic Republic of Ethiopia, 17,187 health posts, 3724 health centers, 302 public hospitals, and 62 private hospitals are found. Based on current data, only 50 of the 90 CT scanners in both public and private health facilities were found to be operational during the study period. Therefore, from January 2023 to March 2024, this study attempted to include every operational multislice CT (MSCT) scanner that was running in Ethiopia's 12 regions and two federal cities with a capability to display dose parameters on its console.

### 2.2. Study Design

To establish national DRLs from all CT facilities accredited and registered by the Ethiopian Technology Authority, a quantitative cross-sectional study design was used. The study excluded centers with operational CT scanners that do not maintain patient data because of storage space constraints and centers with operational CT scanners that experienced data loss from recent repairs. Only 23 health facilities and/or diagnostic centers with functional CT facilities who gave their consent to the study were surveyed, despite the fact that all identified CT facilities that met the inclusion criteria were contacted to participate in the study. As a result, the CT scanners used in this study included one Canon, one Neuviz128; 7 S; eight General Electric; and six Philips. Every location where data were collected has scanners that were produced between 2006 and 2021. Furthermore, the various radiology institutes located throughout the nation have installations that run from 2008 to 2022. The CT scanners that were used to collect the data had variable or fixed tube current settings, automatic exposure control (AEC), and operating tube voltages between 70 and 130 kV. Each CT scanner contained 16–160 slices in total. The study population consisted of all adult patients who visited the MSCT scanners for chest CT examinations.

### 2.3. Sample Size and Sampling Technique

The European Commission specified that data could come from phantoms or standard-size patients. For this reason, standard-size patients were used in this investigation. Restrictions on weight are used to standardize patient sizes. Patients who are extremely big or small are not accepted [25].

Patient dose studies ought to involve a minimum of 20–30 standard-size patients 12. According to available data, the average adult Ethiopian weighs 62 kg in size [9]. All patients with chest CT exams, with the exception of those undergoing specialized CT exams such as perfusion studies or CT angiography and weighing between 40 and 80 kg, were included in order to reach an average weight of 62 kg. A minimum of 35 adult patients were taken from each MSCT scanner that could display dose parameters on its console and whose data were obtained on a CT scanner to have more precision. Of the 50 CT scanners in Ethiopia that had a license and registered by ETA during the study period, only 23 of them agreed to provide the requested data. A total of 801 adult patients whose age above 15 years having accepted image quality of chest CT examination were included to be the study participants.

### 2.4. Data Collection Technique and Statistical Analysis

Radiographers working in the facilities that consented to the study were contacted to assist with data collection. Data were gathered from each healthcare facility using a standardized data collecting format that has been used by the IAEA and international centers for theoretical physics (ICTP). The data were the actual CTDIvol and DLP values displayed on the CT equipment during each examination. The data collection tool also included detailed questions about the age, sex, weight, and body region of each patient. The CT scan parameters were also recorded, including the maker, the CT scanner model, the date of manufacture, the date of installation, the rotation time, the number of detector rows, the beam width, the pitch factor, the number of phases used, and the tube voltage (kV) and current (mA). The data were collected by CT radiographers or radiologic technologists who were recruited from each health facility supervised by the researcher. Radiographers and or radiologic technologists were advised to extract only data of adult patients. Patients were considered adult if they were 15 years or older. In addition, they were advised to collect data of patients whose weight was above 40 kg. The data that are collected were recorded on excel sheets.

For this study, dose data of one of the three common CT examinations, chest CT examination, performed in Ethiopia were extracted. Before entry, data were coded and cleaned for consistency and completeness. We entered and analyzed the data using the Statistical Package for Social Sciences (SPSS) version 26.0. The CT data were analyzed using descriptive statistics. For each anatomic region and CT center, median (50th percentile) and third quartile (75th percentile) of CTDIvol and DLP values were calculated. The third quartile of median CTDIvol and DLP distributions of patient doses, which were derived from a representative sample of the nation's radiology departments, was then used to calculate the national DRLs. The 50th percentile is provided so that institutions whose median dose values are significantly higher than the 75th percentile would evaluate their scan protocol and settings in order to lower patient doses.

### 2.5. Ethical Clearance

The study was approved ethically before it began by the Institutional Review Board of the College of Health Sciences at Addis Ababa University. The CT centers that will take part in the survey gave their consent. Every technique and study protocol used complied with all applicable laws and regulations. In order to ensure privacy and anonymity, the researcher hired data collectors from each diagnostic facility. The investigators were the only ones with access to the data retrieved from the records; personal identification numbers were not utilized.

## 3. Result

As of January 2023, 90 centers in Ethiopia had CT capabilities, according to a preliminary survey, and 40 of those centers had malfunctions at the time of the survey. Just 23 of the study's invited centers submitted dose data. Others either had lost such data from recent repairs or did not keep soft copies of patients and dosage information. The dominant number of the CT systems surveyed was Siemens (5 = 16 slices, 2 = 64 slices, and 1 = 128 slices: *n* = 8). Others included General Electric (GE) scanners (5 = 16 slices and 2 = 64 slices: *n* = 7), Philips scanners (3 = 16 slices, 2 = 64 slices, and 1 = 128 slices: *n* = 6), Canon scanner (160 slices: *n* = 1), and Neusoft scanner (128 slices: *n* = 1).

**Figure 1:**
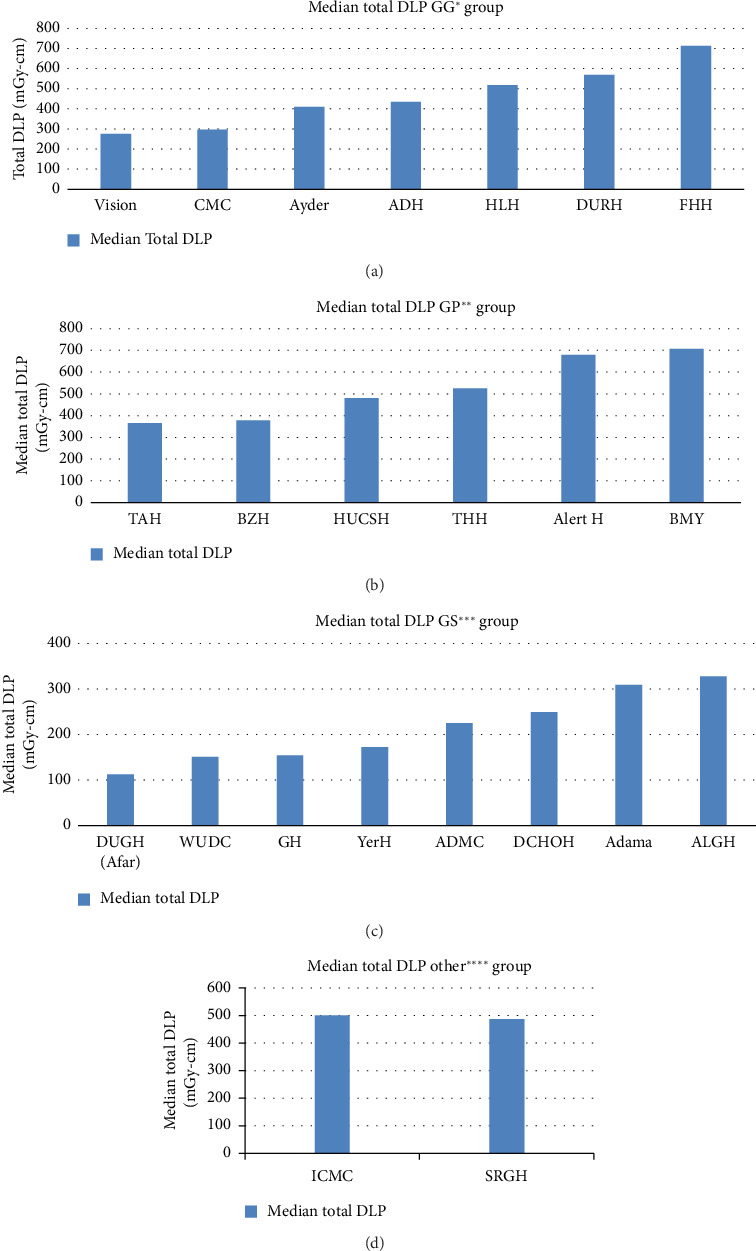
Increasing median DLP distribution for chest adult CT examination using the GG group (a), GP group (b), GS group (c), and other group (d) CT scanners. GG⁣^∗^: general electric CT scanner group, GP⁣^∗∗^: philips CT scanner group, GS⁣^∗∗∗^: siemens CT scanner group, Other⁣^∗∗∗∗^: Canon and Neusoft.

**Figure 2:**
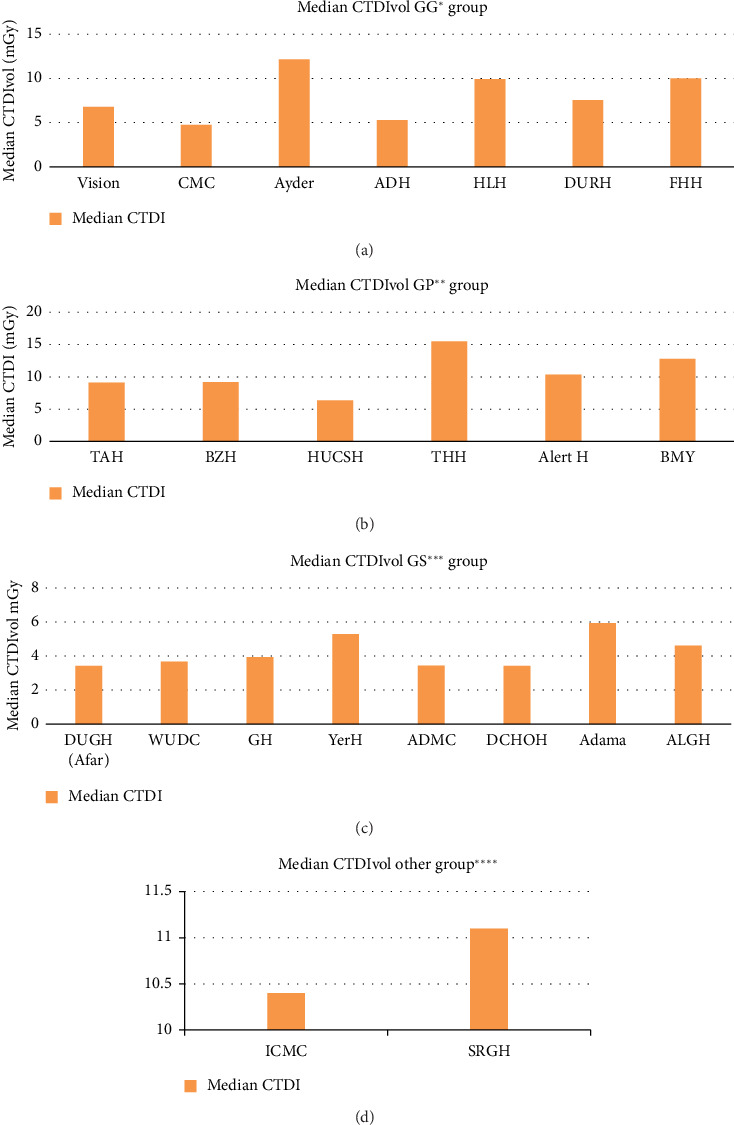
The corresponding median CTDIvol distribution for chest adult CT examination using the GG group (a), GP GP group (b), GS group (c), and other group (d) CT scanners. GG⁣^∗^: general electric CT scanner group, GP⁣^∗∗^: philips CT scanner group, GS⁣^∗∗∗^: siemens CT scanner group, and other group⁣^∗∗∗∗^: Canon and Neusoft.

All identified diagnostic centers were contacted to take part in this study; however, only centers with functional CT facilities that showed willingness to the study were surveyed. So data were collected from 23 health facilities from all over the country. In this study, a total of 801 chest adult patients were investigated during the study period. Of the 801 chest exams performed, 54.81% were on male patients, while 45.19% were on female patients. The mean age of the patients was 48.95 years, with a range of 15–95. The entire data set was utilized from the helical scan mode. The study participants' demographic distribution and characteristics are displayed in Table 1. The CTDIvol and DLP of four groups of CT scanners were compared using descriptive statistics, as indicated in Figures 1 and 2, respectively.

Table 2 displays the dose describers and technical factor median values for all adult patients receiving a CT scan of their chest across all 23 facilities.  Table 3 shows the relationship between CT scan variables and dose indicators. For CTDIvol, there was a weak positive significant relationship with the mAs (*r*_*s*_ = 0.138, *p* value < 0.01), a weak negative significant relationship with the scan length (*r*_*s*_ = −0.109, *p* value < 0.01), and a weak negative significant relationship with the scan time (*r*_*s*_ = −0.132, *p* value < 0.01).

For DLP, there was a weak positive significant relationship with mAs (*r*_*s*_ = 0.118, *p* value < 0.01), with scan time (*r*_*s*_ = 0.168, *p* value < 0.01), with weight (*r*_*s*_ = 0.149, *p* value < 0.01), and with the scan length (*r*_*s*_ = 0.183, *p* value < 0.01). For age and sex, there is no significant relationship for both CTDIvol and DLP cases. Tables 4 and 5 show the relationship between the reference installation year (which we preferred the older year of installation) and the rest of installation years in both total DLP and CTDIvol.  Table 6 presents a comparison between the third quartile values of chest DLP and CTDIvol (mGy) of this study and the NDRL values of a few other published studies.

## 4. Discussion

As indicated in ICRP, population characteristics, technology, and examination protocol can affect DRLs [6]. Therefore, the ICRP has recommended that these factors need to be considered during establishment of DRLs. The current work provides the first Ethiopian NDRLs for chest CT examination. The CT scanner included in this study differs in technology and model. The number of slices per rotation ranges from 16 to 128. The average age of the patients undergoing chest examination was 48.95 years. The majority of the participants were male, with an overall percentage of 54.8%.

It is crucial to analyze scanning methods, evaluate patient dose, and compare with other studies and reference levels in order to accomplish radiation protection for patients [34]. This study found differences in the values of CT dose indicators for adult chest patients across the same median age groups (Table 2). For example, for both GP and GS CT scanners groups having similar median age groups of 38–53 years and 38–54.5 years, they have median TDLP of 378.3–707.5 mGy.cm and 151–327.59 mGy.cm, respectively. This difference indicates that dose indicators are independent of ages of the patients (Table 3). Our study's demonstrated that the CT scanners' doses varied significantly from one another (Figures 1 and 2). The difference in scanning procedures, chest CT clinical indications, type of scanners, radiographer training, and experience can all lead to dose variations. Radiation doses for CT examinations vary considerably among patients and facilities. This difference is mostly ascribable to the technical parameters of the CT scanning protocols. This variability underscores the need for standardizing imaging protocols to improve patient safety, reduce unnecessary radiation exposure, and optimize imaging parameters. An optimization process is a team effort. Thus, staff training is highly important to enhance dose optimization.

Even though all health facilities were using dose optimization strategies, such as tube current modulations, which can dramatically minimize patient exposure, variability of CT dose indicators was also observed from the same CT scanner, model, and anatomical location. The ranges of scan parameters for chest CT examinations collected in the survey are shown in Table 2. Significant variation was noted in all parameters, except for kVp. A tube voltage of 120 kV was used in nearly all CT procedures. These differences could be the consequence of manufacturer-specific changes in CT equipment design or user choices of settings for the same anatomical location. For example, the highest variation of total DLP for almost equal mean weight was seen in GG CT scanner groups between FHH and vision CT scanner with median values of 704 (mGy-cm) and 274.86 (mGy-cm) (Figure 1, Table 1). This is primarily related to increased usage of mAs and CTDIvol (Table 2). Similarly, the smallest variation of median values of DLP was seen in the GS CT scanner groups between DUGH and ALGH with a maximum to minimum ratio of 2.68 for median weight of 56.5 kg and 61 kg (Table 1), respectively. The main causes of these small differences in GS CT scanners are its small variation in the usage of sequence, kVp, scan range, and CTDI with the corresponding values of 1.5, 1.18, 1.02, and 1.3, respectively (Tables 1 and 2).

When we compared our CTDIvol estimates with those of Ugandan 2022 publications [26], there was a weak and moderate positive significant relationship with the mAs examination (*r*_*s*_ = 0.118, *p* value < 0.01) and (*r*_*s*_ = 0.7372, *p* value < 0.001), respectively. Similarly, both our studies and Ugandan studies have a weak negative significant relationship with the scan length (*r*_*s*_ = −0.183, *p* value < 0.01) and (*r*_*s*_ = −0.2918, *p* value = 0.0029), respectively. For DLP, both the current study and Ugandan 2022 studies have weak positive significant relationship with the mAs examination (*r*_*s*_ = 0.118, *p* value < 0.01) and (*r*_*s*_ = 0.4869, *p* value < 0.001), respectively (Table 3). Although we could not find research conducted regarding different ages of CT scanners, our current study in Tables 4 and 5 shows inconsistent relationship between the reference installation year (which we preferred the older year of installation) and the rest of installation years in both total DLP and CTDIvol.

We have compared the proposed NDRLs of the current study with other established NDRLs in Uganda (2022) [26], Nigeria (2018) [21], Australia (2020) [27], Japan (2020) [28], the UK (2019) [22], Ghana (2024) [29], Egypt (2017) [30], and other selected studies (Table 3). Even though the CTDIvol of the current study is less than most African and non-African studies mentioned in the comparison table, when comparing the current NDRLs to those of African nations, the adult 75th percentile of DLP values of our results was less than Nigeria (2018) [21] and Kenya (2016) [31] but greater than Uganda (2022) [26, 31] and Egypt (2017) [30] published studies. The work from Ghana (2024) [29] displayed the largest deviation compared to the current investigations (509.12.mGy.cm) (Table 6).

Similarly, when the present study was compared with other non-African countries, the proposed NDLP of the current study was substantially higher than other similar studies conducted in Australia (2020) [27], Turkiye (2015) [32], Switzerland (2020) [33], and the UK (2019) [22] (Table 6). This gap could be explained by the number of sequences, scan parameters, scan time, IR, and AEC, all of which improve optimization and boost scanner dosage efficiency [34] for non-African nations.

To enable further reduction in the CT radiation dose used in Ethiopia, this study recommends the first national DRLs on one of the common CT examinations for adult patients in Ethiopia based on a nationwide survey using a variety of MSCT scanners. Even though additional training on appropriate CTDIvol and DLP usage may be necessary for radiologists and radiographers, the establishment of NDRLs will significantly reduce the amount of CT radiation used in Ethiopia.

### 4.1. Limitations

The current study is not without limitations. We have to admit that there could be two possible drawbacks in our study. First, the comparison we made on our proposed national DRLs of CTDIvol and DLP with other published studies is without weight consideration. Secondly, the study compared the median CTDIvol and DLP values across different health facilities without taking into account the particular clinical indications for the chest CT examinations or the type of reconstruction used. Exam classification is based solely on the body part examined, not on the extra data needed to determine the clinical indication for the imaging exam. This limitation may compromise the accuracy of the comparisons, as different clinical indications may require different levels of radiation dose, and different reconstruction methods can influence the dose values.

## 5. Conclusion

The results show significant variations in dosage both within and between Ethiopian centers. Additionally, the CTDIvol is comparable to the international standard, but the DLP is significantly higher. The authors of this study conclude that, the findings of this research will enable the health facilities to compare their patient doses with national benchmarks. The new NDRLs will help facilities effectively optimize their CT protocols for the wide range of patient sizes they examine. It allows them to appropriately reduce patient doses because smaller patients require lower doses than larger ones to yield adequate image quality.

High doses and dose variations seem to be significantly influenced by technical and technological factors. A decrease in dose variations and median dose levels should be facilitated by improvements in CT technology and protocol optimization, including exposure and technical parameter selection. Centers with large dose variations should think about standardizing protocols to narrow dose values. Centers with dose outliers, defined as values above the 75th percentile, urgently need to investigate as low as reasonably achievable dose protocols.

Our findings provide a national point of reference for CT doses and have a duty to expedite optimization strategies to reduce the dose burden from chest CT examinations across Ethiopia. It is also helpful for policy formulation, monitoring and evaluation activities of the government and different concerned agencies, and communities at large. To enhance the radiation protection program, a corrective dose reduction strategy needs to be designed without affecting image quality. Adopting dose-reduction strategies, such as adjusting exposure parameters such as tube current (mA) and tube potential (kVp) and optimizing scan range, is one of the main recommendations for reducing radiation exposure. This can be accomplished by giving CT staff members sufficient training on the variables influencing patient dosage and image quality, choosing the best scanning parameters, and carefully selecting the anatomical region by reducing scan lengths to only include the anatomical region that are required. The current study's findings provide insight into the radiation dosages adult patients in Ethiopia health facilities experienced during chest CT examinations. Moreover, in the case of adult chest CT exams, a comparable kind of extensive survey based on clinical indication ought to be carried out to establish national DRLs.

## Figures and Tables

**Table 1:** Demographic distribution and characteristics of study participants: chest.

Facilities	Sex		Age (years)		Weight (kg)		
	M *n* (%)	F *n* (%)	Range (Min, Max)	Mean ± SD	Range (Min, Max)	Mean ± SD	Median
ADH	19 (54.30)	16 (45.70)	59 (17, 76)	43.69 ± 15.64	20 (52, 72)	60.89 ± 4.78	59.00
AdamaG	15 (57.10)	13 (42.9)	45 (19, 64)	38.29 ± 15.78	28 (52, 80)	64.07 ± 6.71	64.00
ADMC	23 (65.7)	12 (34.3)	64 (22, 86)	55.60 ± 16.17	37 (43, 80)	58.94 ± 11.67	58.00
DUGH (Afar)	22 (64.7)	12 (35.3)	63 (17, 80)	45.41 ± 17.25	33 (43, 76)	58.61 ± 8.92	56.00
ALGH	21 (60)	14 (40)	60 (20, 80)	42.71 ± 16.63	39 (40, 79)	59.64 ± 10.91	61.00
AlertH	16 (45.7)	19 (54.3)	72 (18, 90)	45.09 ± 19.02	23 (46, 69)	58.2 ± 6.63	59.00
Ayder	20 (57.1)	15 (42.9)	63 (22, 85)	59.29 ± 14.82	35 (40, 75)	52.03 ± 10.15	52.00
BZH	17 (48.6)	18 (51.4)	66 (20, 86)	47.29 ± 15.08	29 (51, 80)	67.66 ± 7.8	69.00
BMYl	21 (58.3)	15 (41.7)	67 (23, 90)	55.31 ± 19.15	40 (40, 80)	62.31 ± 11.53	60.00
DURH	23 (65.7)	12 (34.3)	78 (17, 95)	45.11 ± 20.16	26 (48, 74)	62.86 ± 6.18	62.00
DCHOH	15 (42.9)	20 (57.1)	55 (25, 80)	50.46 ± 16.27	40 (40, 80)	60.94 ± 10.33	60.00
FHH	21 (60)	14 (40)	51 (19, 70)	47.63 ± 15.92	39 (41, 80)	61.51 ± 9.31	62.00
GH	16 (50)	16 (50)	51 (25, 76)	51.69 ± 17.03	40 (40, 80)	62.77 ± 11.35	61.00
HLH	21 (60)	14 (40)	60 (25, 85)	50.31 ± 16.93	32 (48, 80)	66.6 ± 8.58	68.00
HUCSH	20 (57.1)	15 (42.9)	72 (18, 90)	45.6 ± 19.4	35 (45, 80)	60.23 ± 10.40	56.00
ICMC	19 (54.3)	16 (45.7)	61 (19, 80)	56.03 ± 19.58	27 (47, 74)	61.77 ± 7.58	60.00
CMC	20 (57.1)	15 (42.9)	55 (28, 83)	56.89 ± 17.66	39 (41, 80)	63.43 ± 12.01	65.00
ASRG	25 (71.4)	10 (28.6)	72 (15, 87)	44.49 ± 18.68	32 (48, 80)	69.83 ± 8.22	70.00
TASH	14 (40)	21 (60)	63 (17, 80)	42.66 ± 15.84	33 (45, 78)	58.51 ± 9.28	58.00
THH	20 (57.1)	15 (42.9)	63 (17, 80)	48.94 ± 18.32	36 (44, 80)	62.40 ± 10.69	62.00
Vision	11 (31.4)	24 (68.6)	58 (24, 82)	54.14 ± 16.25	34 (43, 77)	59.43 ± 8.91	61.00
WUDC	13 (37.1)	22 (62.9)	64 (18, 82)	48.69 ± 18.62	33 (42, 75)	53.83 ± 9.07	52.00
YerH	21 (60)	14 (40)	54 (28, 82)	50.66 ± 14.17	31 (49, 80)	67.37 ± 9.33	69.00
Total	433 (54.5)	362 (45.5)	80 (15, 95)	48.95 ± 17.15	40 (40, 80)	61.47 ± 9.15	61.00

**Table 2:** Median, range values of technical factors and dose describers of all adult patients undergoing chest CT examination in all twenty three hospitals.

CT scanner groups	Facilities (hospitals)	Age	(Wtkg)	Sequence	kV (min, max)	mA	mAs (min, max)	SR (cm) (min, max)	MCTDIvol	TDLP (mGy.cm)	Installation year of CT
GP	AlertH	40	58.5	2	120 (120, 120)	326.53	161 (103, 241)	35.2 (28.7, 41.6)	10.33	679.65	2015
	BZH	47	69	1	120 (120, 120)	182.67	137 (81, 295)	33 (30, 35)	9.2	378.3	2022
	BMY	55	60	2	120 (120, 120)	250	190 (106, 235)	30 (28, 31)	12.77	707.5	2023
	HUCSH	38	60	4	120 (120, 120)	260	130 (65, 140)	34.8 (28.5, 43.4)	6.39	481.1	2013
	TAH	38	58	1	120 (120, 120)	285	142.5 (75, 318)	40.3 (38, 42.5)	9.145	365.8	2008
	THH	53	62	2	120 (120, 120)	375	200 (200, 265)	40 (39, 43)	15.5	524.8	2018

GS	AdamaG	38	64	2	130 (130, 130)	72	60 (32, 137)	32.4 (20.1, 51.5)	5.94	309.21	2022
	ADMC	44	60	2	120 (120, 120)	139	32 (19, 86)	24.95 (22, 43)	3.43	225	2021
	DUGH (Afar)	40	56.5	1	120 (120, 120)	55.315	32.5 (19, 86.25)	32.7 (15.7, 45.6)	3.42	112.5	2022
	ALGH	40	61	3	130 (130, 130)	80.67	54 (52, 58)	42.2 (31.8, 52.4)	4.61	327.59	2020
	DCHOH	50	60	2	80 (70, 110)	131	123 (48, 210)	33.7 (23, 41)	3.41	249	2022
	GH	54.5	61	1	110 (100, 110)	63	63 (37, 209)	30.96 (26, 38.7)	3.93	154.5	2018
	WUDC	48	52	2	120 (120, 120)	145.45	48 (28, 232)	34.6 (23, 43.9)	3.67	151	2019
	YerH	49	69	1	130 (130, 130)	57	54 (51, 57)	45 (42, 47)	5.28	172	2020

GG	ADH	44	60	2	120 (120, 120)	139	94 (70, 250)	24.95 (11, 50.6)	5.28	433.7	2018
	Ayder	60	52	1	120 (120, 120)	200	200 (118, 440)	29 (20.5, 49.5)	12.16	409.6	2013
	DURH	45	62	2	120 (120, 120)	154.5	123.6 (79, 246)	36.5 (23, 41.4)	7.54	567.7	2021
	FHH	50	62	2	120 (120, 120)	115	124 (117, 126)	33 (20, 55)	10	713	2016
	HLH	49	68	2	120 (120, 120)	200	200 (195, 206)	31 (19.3, 44)	9.91	517.24	2017
	CMC	59	65	2	120 (120, 120)	82	65.6 (64, 201)	31.4 (25, 38)	4.73	295.66	2015
	Vision	58	61	2	120 (120, 120)	100	100 (64, 128)	34.513 (11, 55)	6.8	274.86	2020

Other	ASRG	64	60	2	120 (120, 120)	250	198 (190, 216)	35 (30, 39.5)	10.4	501	2019
	ICMC	42	70	2	120 (120, 120)	270	180 (177, 306)	35 (25, 50)	11.1	486.7	2021

*Note:* GG: general electric CT scanners group; GP: Philips CT scanners group; GS: Siemens CT scanners group; other: Canon and Neusoft.

**Table 3:** Spearman rank correlation coefficients for the relationship between continuous CT scan variables and diagnostic reference level estimates.

rs (*p* value)	mAs	Scan length (mm)	Scan times	Weight	Age	Sex
CTDIvol mGy	0.138 (0.001)⁣^∗^	−0.109 (0.002)⁣^∗^	−0.132 (0.005)⁣^∗^	−0.013 (0.718)	0.006 (0.875)	0.001 (0.969)
TDLP mGy.cm	0.118 (0.003)⁣^∗^	0.183 (0.000)⁣^∗^	0.168 (0.00)⁣^∗^	0.149 (0.00)⁣^∗^	−0.02 (0.587)	0.095 (0.056)

⁣^∗^Significant relationship.

**Table 4:** The relationship between different types and installation year of the CT scan machine with total DLP diagnostic reference level estimates.

Coefficients^a^						
Installation year		Unstandardized coefficients		Standardized coefficients	*t*	Sig.
		*B*	Std. error	Beta		
Model Philips^b^	(Constant)	480.949	56.094		8.574	0
	2022	118.827	68.539	0.165	1.734	0.084
	2018	105.139	68.7	0.146	1.53	0.127
	2015	301.404	79.91	0.326	3.772	0

Model Siemens^c^	(Constant)	318.773	57.892		5.506	0
	2022	−19.526	67.968	−0.029	−0.287	0.774
	2021	−51.201	77.67	−0.059	−0.659	0.51
	2020	−44.315	77.67	−0.051	−0.571	0.569
	2018	−121.318	79.866	−0.133	−1.519	0.13
	2012	−151.23	77.67	−0.175	−1.947	0.053

Model GE^b^	(Constant)	475.309	50.48		9.416	0
	2021	175.323	71.39	0.187	2.456	0.015
	2020	−137.934	71.39	−0.147	−1.932	0.055
	2018	76.103	71.39	0.081	1.066	0.287
	2017	215.485	61.975	0.295	3.477	0.001
	2015	−147.853	−147.853	−0.158	−2.071	0.039

^a^Dependent variable: total DLP.

^b^Reference installation year: 2013.

^c^Reference installation year: 2008.

**Table 5:** The relationship between different types and installation year of the CT scan machine with CTDIvol diagnostic reference level estimates.

Coefficients^a^						
Installation year		Unstandardized coefficients		Standardized coefficients	*t*	Sig.
		*B*	Std. error	Beta		
Model: Philips^b^	(Constant)	6.539	0.681		9.599	0
	2022	5.139	0.832	0.53	6.174	0
	2018	6.652	0.834	0.684	7.973	0
	2015	4.029	0.963	0.327	4.182	0

Model: Siemens^c^	(Constant)	6.385	21.505		0.297	0.767
	2022	89.888	24.179	0.363	3.718	0
	2021	−2.596	28.851	−0.007	−0.09	0.928
	2020	−0.506	28.851	−0.001	−0.018	0.986
	2018	−1.928	29.667	−0.005	−0.065	0.948
	2012	−2.424	28.851	−0.007	−0.084	0.933

Model: GE^b^	(Constant)	475.309	50.48		9.416	0
	2021	175.323	71.39	0.187	2.456	0.015
	2020	−137.934	71.39	−0.147	−1.932	0.055
	2018	76.103	71.39	0.081	1.066	0.287
	2017	215.485	61.975	0.295	3.477	0.001
	2015	−147.853	71.39	−0.158	−2.071	0.039

^a^Dependent variable: mean CTDIvol.

^b^Reference installation year: 2013.

^c^Reference installation year: 2008.

**Table 6:** Comparison of the established national DRLs of this study with other selected African and non-African national diagnostic reference levels.

CT dose describers	This study Ethiopia	Uganda (2022) [26]	Nigeria (2018) [21]	Australia (2020) [27]	Japan (2021) [28]	The UK (2019) [22]	Ghanna (2024) [29]	Egypt (2017) [30, 31]	Kenya (2015) [31]	Turkiye (2015) [32]	Switzerland (2020) [33]
CTDIvol (mGy)	**10.165**	7.8	17	10	13	8.5	15.7	22	19	11.6	7
DLP (mGy-cm)	**509.12**	377	735	390	510	290	1102.8	420	895	289	250

*Note:* Bold values indicate the values of the current study.

## Data Availability

All the necessary data and materials have been included in this manuscript.
